# Subinhibitory Antibiotic Concentrations Mediate Nutrient Use and Competition among Soil *Streptomyces*

**DOI:** 10.1371/journal.pone.0081064

**Published:** 2013-12-05

**Authors:** Patricia Vaz Jauri, Matthew G. Bakker, Christine E. Salomon, Linda L. Kinkel

**Affiliations:** 1 Department of Plant Pathology, University of Minnesota, Twin Cities, Minnesota, United States of America; 2 Center for Drug Design, University of Minnesota, Twin Cities, Minnesota, United States of America; J. Craig Venter Institute, United States of America

## Abstract

Though traditionally perceived as weapons, antibiotics are also hypothesized to act as microbial signals in natural habitats. However, while subinhibitory concentrations of antibiotics (SICA) are known to shift bacterial gene expression, specific hypotheses as to how SICA influence the ecology of natural populations are scarce. We explored whether antibiotic ‘signals’, or SICA, have the potential to alter nutrient utilization, niche overlap, and competitive species interactions among *Streptomyces* populations in soil. For nine diverse *Streptomyces* isolates, we evaluated nutrient utilization patterns on 95 different nutrient sources in the presence and absence of subinhibitory concentrations of five antibiotics. There were significant changes in nutrient use among *Streptomyces* isolates, including both increases and decreases in the capacity to use individual nutrients in the presence vs. in the absence of SICA. Isolates varied in their responses to SICA and antibiotics varied in their effects on isolates. Furthermore, for some isolate-isolate-antibiotic combinations, competition-free growth (growth for an isolate on all nutrients that were not utilized by a competing isolate), was increased in the presence of SICA, reducing the potential fitness cost of nutrient competition among those competitors. This suggests that antibiotics may provide a mechanism for bacteria to actively minimize niche overlap among competitors in soil. Thus, in contrast to antagonistic coevolutionary dynamics, antibiotics as signals may mediate coevolutionary displacement among coexisting *Streptomyces*, thereby hindering the emergence of antibiotic resistant phenotypes. These results contribute to our broad understanding of the ecology and evolutionary biology of antibiotics and microbial signals in nature.

## Introduction

Antibiotic-producing bacteria are common in natural habitats, yet the roles of antibiotics in the ecology and evolutionary biology of soil populations remain poorly understood. While traditionally perceived as weapons mediating competitive interspecies interactions [1], recent work emphasizes a role for antibiotics as signals [2]–[4]. It has been argued that antibiotics are rarely present at inhibitory concentrations in the environment, and thus that their role must be mediated primarily by whatever functions they accomplish at subinhibitory concentrations. However, despite significant evidence that subinhibitory concentrations of antibiotics (SICA) can alter bacterial gene expression in vitro, thus acting as signals [5]–[10], there is little experimental support from natural habitats for a predominant role for antibiotics as either signals or weapons. Our lack of understanding of the specific roles of antibiotics in natural populations constrains our abilities to identify habitats or selective conditions most likely to generate novel antibiotic phenotypes and to predict the dynamics of antibiotic resistance in environmental microbes. As weapons, antibiotics are assumed to mediate antagonistic interactions in soil [11], and antibiotic-producing microbes have been suggested to exhibit coevolutionary arms race dynamics [12]. The arms race model predicts reciprocal accumulation of matching antibiotic inhibitory and resistance capacities in interacting populations over time, with ongoing selection for resistance in the presence of antibiotic-producers. In contrast, antibiotics as signals have been hypothesized to mediate neutral, or even cooperative species interactions [9], [13]–[14]. If antibiotics mediate signaling interactions, there may be little reason to expect ongoing selection for resistance, suggesting the potential for fundamentally different coevolutionary dynamics. However, specific mechanisms by which antibiotic signals may facilitate non-antagonistic interactions remain predominantly hypothetical.

Among antibiotic-producing microbes, the *Streptomyces* (Order Actinomycetales, Family Streptomycetaceae) are notable as prolific producers of antibiotics [15]–[16]. *Streptomyces* are gram-positive, filamentous bacteria, excellent saprophytes, and ubiquitous in soil [17]. Because nutrient competition is a primary driver of species interactions in soil [18], this work considers the hypothesis that SICA play a significant role in mediating nutrient competition among soil *Streptomyces*. Recent work shows that antibiotic inhibition is greatest among sympatric *Streptomyces* that have large niche or nutrient use overlap (Kinkel *et al.*, in press), supporting a role for antibiotics as weapons in nutrient competition. However, does the role of antibiotics in mediating species interactions change when antibiotics are present at subinhibitory concentrations?

The goal of this work is to evaluate the potential for SICA to alter microbial phenotypes in ways that may significantly influence species interactions. The effects of SICA on nutrient use, niche overlap, and escape from competition were evaluated for a collection nine *Streptomyces* isolates. SICA shifted nutrient use in all isolates. Nutrient overlap among pairwise combinations of isolates was often reduced. More significantly, because of SICA-induced shifts in nutrient use among isolates, the proportion of growth on all nutrients that were not used by a competitor frequently increased in the presence as compared with the absence of SICA when considering all pairwise isolate combinations. These data suggest that SICA may in some cases mediate niche differentiation among soil microbes, thus minimizing the potential for competitive conflicts among coexisting microbes, and consequently reduce selection for both antibiotic resistance and enhanced antagonistic phenotypes.

## Materials and Methods

### Bacterial Isolates

The *Streptomyces* isolates used were collected from diverse natural habitats: 1232.2, 3211.5, and 5111.5 were from a prairie in the central USA, Cev2-10 was from a rocky hillside in southwestern France, Lub2-11b was from a forested area in south-central France, Mont3-8 was from a forest in eastern Spain, NZ816-12 was from a beach forest on the South Island of New Zealand, Pan FS14 was from a forested site in central Panama, and Witz25 was from a forested hillside in west-central Germany (Table S1). The work was non-destructive and had no impacts on local sites, and thus for most sites permits were not required. The governments of Panama and New Zealand issued permits for sampling in their reserved areas. Isolates used here were selected from a global collection based on their diversity in nutrient utilization patterns (data not shown). Analysis of 16S sequences documented that all isolates are members of the genus *Streptomyces* (data not shown).

### Antibiotic Treatments

Five antibiotics produced by or derived from Actinobacteria were used in this work, including rifampicin, tetracycline, vancomycin, streptomycin and chloramphenicol [15]. Minimum inhibitory concentrations (MIC) were determined for each isolate-antibiotic combination on solid ISP2 medium [19] using filter-sterilized antibiotic stocks [20]. Antibiotics were incorporated into ISP2 medium at a range of concentrations, and four individual 4 µl drops of suspensions of 1×10^8^ spores/ml of the same isolate were spotted onto each plate. Plates were incubated at 28°C and presence/absence of growth was observed after 3 days. The MIC was determined as the lowest concentration that prevented any detectable growth among replicate spots. The subinhibitory concentration of each antibiotic (SICA) was subsequently defined as 10% of the MIC for each antibiotic-isolate combination. Growth of each isolate on the subinhibitory concentration for each antibiotic was confirmed and compared to growth on non-amended ISP2 medium. For every isolate-antibiotic combination, we confirmed the presence of vigorous growth on solid ISP2 medium amended with the subinhibitory concentration of each antibiotic.

### Nutrient Use

Nutrient utilization by each isolate in each condition was determined as reported previously [21]. Briefly, Biolog SF-P2 plates (Biolog, Inc. Hayward, CA) were inoculated as indicated by the manufacturer (http://www.biolog.com). Solutions with the corresponding SICA were mixed with spore suspensions of each isolate immediately prior to inoculation of Biolog plates to reach a final OD 590 of 0.22. Following inoculation, plates were incubated for 3 days at 28°C, and growth on each of the n = 95 nutrients was determined as OD 590 of each well using a Multiskan EX microplate reader (Labsystems, Helsinki, Finland). Growth on Biolog plates SF-P2 is measured by direct quantification of optical density rather than a metabolic dye. For each plate, the O.D. of the water control well was subtracted from that of all other wells prior to analysis, and differenced O.D. values below 0.005 were considered as no growth. Nutrient use was evaluated on three replicate plates for every isolate-antibiotic combination. Total growth was calculated as the sum of the absorbance values on all nutrients and niche width was the number of nutrients for which absorbance values were positive.

The effect of SICA on nutrient competition was first evaluated through the calculation of the nutrient overlap among all possible isolate-isolate combinations in all SICA. Nutrient overlap (NO) for each isolate-isolate-antibiotic combination was calculated using the formula:
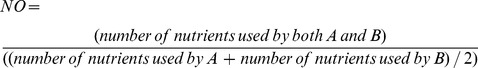



Since reductions in NO were frequently associated with reductions in total activity of isolates under SICA conditions, we defined a new measure of competition, the escape ratio (ER). The ER was defined as the ratio of competition-free growth in the presence of SICA to the competition-free growth in control conditions. Thus, when ER>1, the presence of SICA could potentially reduce competition among isolates in comparison to control conditions. As for NO, the ER was determined for every isolate in combination with every other isolate in the presence and absence of antibiotics using the formula:
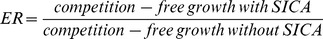
where competition-free growth is the sum of the growth that occurred on all nutrients that were not utilized by the paired isolate.

### Data Analyses

Student t-tests used to determine significance of shifts in nutrient use and ANOVAs and Tukey’s LSD tests to determine shifts in total activity and niche width were carried out using Matlab Statistics Toolbox (MATLAB version 7.8.0. Natick, MA: The MathWorks Inc., 2009). Chi-square tests to determine differences in frequencies of ER>1 among antibiotics were carried out using an online interactive calculation tool for chi-square goodness of fit (http://quantpsy.org).

## Results and Discussion

**Figure 1:**
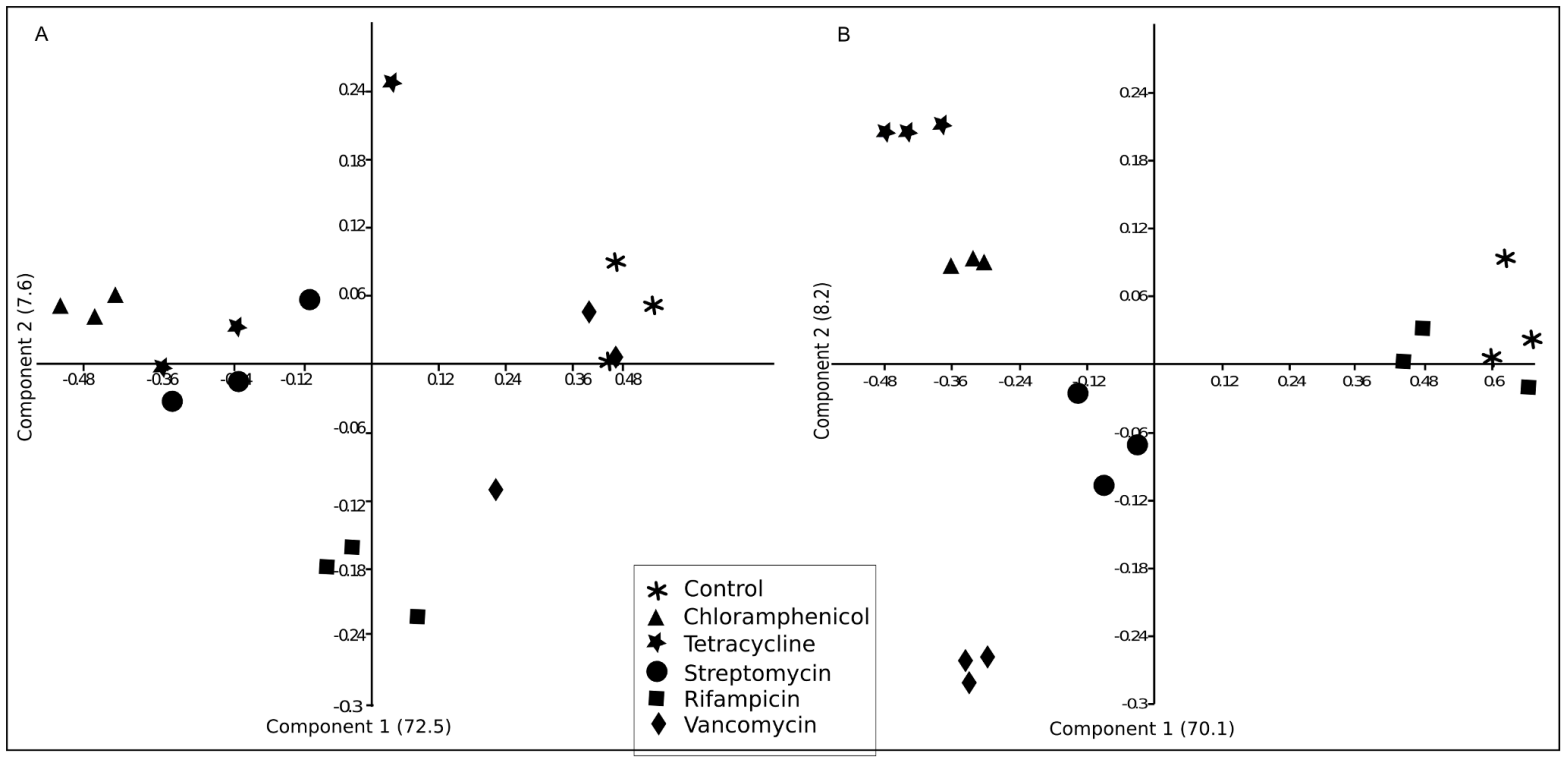
Principal components analysis of nutrient use profiles of *Streptomyces* isolates. Principal components analysis of nutrient use profiles for *Streptomyces* isolates 1232-2 and Mont3-8 in the presence and absence of SICA. Growth was evaluated on Biolog SF-P2 plates; each treatment was replicated three times. The percent variation in nutrient use explained by each axis is shown in parentheses.

Nutrient use among *Streptomyces* was significantly influenced by SICA. Shifts in nutrient use in response to SICA were consistent among replicates for each isolate-antibiotic combination, but varied widely among isolates and antibiotics (Figure 1). Among isolates, total growth (growth summed over all nutrients) was significantly reduced in the presence of SICA in one-third of isolate-antibiotic combinations (Table 1; ANOVA; p<0.05). Among antibiotics, chloramphenicol and tetracycline had the most consistent negative impacts on total growth. Despite the lack of evidence for negative impacts of each SICA on the growth of isolates on complex media, every SICA had targeted impacts on growth on specific nutrients, including both increases and decreases in growth over 3 days.

**Table 1:** Origin, total growth on all nutrients, niche width, and changes in niche width in the presence and absence of SICA.

		TOTAL GROWTH (O.D.)		NICHE WIDTH (N. Nut.)		CHANGES IN NICHE WIDTH WITH SICA (% change)				
ISOLATE	ORIGIN	W/O SICA	WITH SICA	W/O SICA	WITH SICA	CHLOR	TET	STREP	RIF	VANC
1232-2	Minnesota, USA	6.86	3.30 (*)	81	41 (*)	−75.4 (*)	−37.7	−56.6 (*)	−62.3 (*)	−16.0
3211-5	Minnesota, USA	4.97	4.01	59	66	−16.5	12.5	19.9	8.0	37.5
5111-5	Minnesota, USA	3.41	1.74	41	37	−82.3 (*)	−33.1	17.7	45.1	0.8
Cev2-10	Cevennees, France	8.28	6.63	80	65 (*)	−20.0 (*)	−29.6 (*)	−13.3	−6.3	−24.2 (*)
Lub2-11b	Luberon, France	2.98	2.49	69	64	−17.4	1.0	−25.6	−15.9	24.2
Mont3-8	Montseny, Spain	10.87	6.85 (*)	70	67	−65.9	129.4	34.1	63.5	34.1
NZ816-12	New Zealand	0.89	1.18	28	39	−0.5	−11.4	−0.5	−8.6	−2.9
PanFS14	Fort ShermanPanama	2.63	1.69	48	37	−14.5	−60.7	−46.9	−2.8	4.1
Witz25	WitzenhausenGermany	4.84	3.48	50	45	−30.0	2.7	12.1	−2.0	−30.2

Origin, total growth on all nutrients, niche width (number of nutrients used), and changes in niche width in the presence and absence of SICA. Values for total growth and niche width with SICA are the means for each isolate among all five antibiotic treatments. Changes in niche width are reported as percent change in total growth in the presence vs. the absence of each antibiotic. Significant differences in total growth or niche width in the presence and absence of antibiotics are indicated by (*) (t-test, p<0.05). O.D.: optical density; N. nut.: number of nutrients; CHLOR: chloramphenicol; TET: tetracycline; STREP: streptomycin; RIF: rifampicin; VANC: vancomycin.

Tetracycline, vancomycin and rifampicin induced the highest frequency of significant increases in growth on particular nutrients in the presence versus the absence of antibiotics, increasing growth in 2% (tetracycline) and 1.4% (vancomycin, rifampicin) of 855 nutrient-isolate combinations (data not shown). Considering both increases and decreases in growth, chloramphenicol had the greatest impact on nutrient use, shifting growth significantly on 22% of 95 nutrients on average among isolates. In contrast, rifampicin had relatively little effect on nutrient use, with significant impacts on use of only 8.7% nutrients on average among isolates.

*Streptomyces* isolates varied in their growth responses to the same antibiotic. For example, streptomycin increased growth of isolate Cev2-10 on N-acetyl-D-glucosamine, but decreased growth of isolates 1232-2 and 5111-5 on the same nutrient. For some isolates, growth on a particular nutrient was especially sensitive to antibiotics. For instance, growth of 1232-2 on putrescine was reduced significantly by subinhibitory concentrations of every antibiotic tested, but growth on putrescine was unchanged for all other isolates in the presence vs. absence of any SICA. Perhaps most surprisingly, some isolates grew on specific nutrients only when antibiotics were present. For example, Lub2-11b was able to grow on both N-acetyl-β-D-mannosamine and α-D-lactose in the presence of subinhibitory concentrations of vancomycin and tetracycline but not in the antibiotic-free control (t-test = 7.6 and 4.43, respectively, p<0.02).

Because SICA both eliminated growth on some nutrients and induced growth on others, SICA modified *Streptomyces* niche width, the total number of nutrients utilized by an isolate (Table 1, Table S2). Niche widths of individual isolates were significantly reduced in the presence vs. absence of SICA in only 7 isolate-antibiotic combinations (**t-test**, p<0.05; Table 1). Six of these involved the same 2 isolates (1232-2 and Cev2-10) which appear to be especially sensitive to SICA.

Isolate-specific shifts in nutrient use and niche width suggest the potential for SICA to influence nutrient competition among *Streptomyces*. Consequently, niche overlap (NO) among all pairwise isolate combinations in the presence and absence of SICA were calculated. Antibiotics reduced NO relative to the control in 56% (146) of the 360 possible isolate-isolate-antibiotic combinations, with reductions ranging from 0.01% to 91% (mean reduction = 28%). Increases in NO ranged from 0.2% to 340% (mean increase = 41.7). However, because total growth was sometimes concurrently reduced by SICA, reductions in NO may be of limited benefit to *Streptomyces* fitness overall. Although a reduced nutrient overlap may suggest there is less competition for nutrients, if total growth of the isolate is significantly reduced in the presence of SICA, the conditions are unlikely to increase the fitness of the isolate.

**Figure 2:**
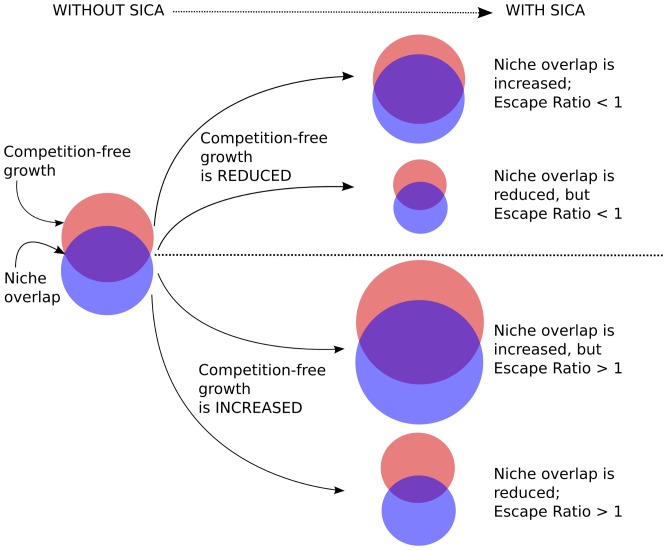
Characterizing Escape Ratios (ER) among competing microbes. The niche of each microbe is denoted by a shaded circle, which represents the cumulative growth of an isolate on the total suite of nutrients used by that isolate. Niche overlap is the area of intersection between the two circles, and quantifies the total growth for each isolate on nutrients used by both isolates. Competition-free growth is the non-intersecting area for each circle, or total growth for an isolate on nutrients that the competing isolate does not use. The escape ratio (ER) is the ratio of competition-free growth in the presence vs. the absence of SICA. When the escape ratio is greater than one, we hypothesize benefits to an isolate from the presence of SICA.

As an alternative for characterizing effects of SICA on nutrient competition, we calculated the total competition-free growth for each isolate, or cumulative growth for an isolate on all nutrients that were not utilized by a paired isolate, for all pairwise isolate combinations on each antibiotic (Figure 2). Competition-free growth provides a measure of escape from competition, and may be either increased or decreased by SICA. These increases or decreases can be indexed by an ‘escape ratio’, or the ratio of the total competition-free growth in the presence vs. the absence of SICA. Escape ratios greater than one characterize isolate-antibiotic combinations in which there are increases in total growth that are competition-free in the presence of SICA. Escape ratios greater than one suggest SICA-isolate combinations in which isolates have undergone specific shifts in nutrient utilization in response to SICA that reduce the impacts of nutrient competition on total growth. In this case, SICA may act to enhance the fitness of interacting isolates, or reduce the negative impacts of resource competition on competitors.

**Figure 3:**
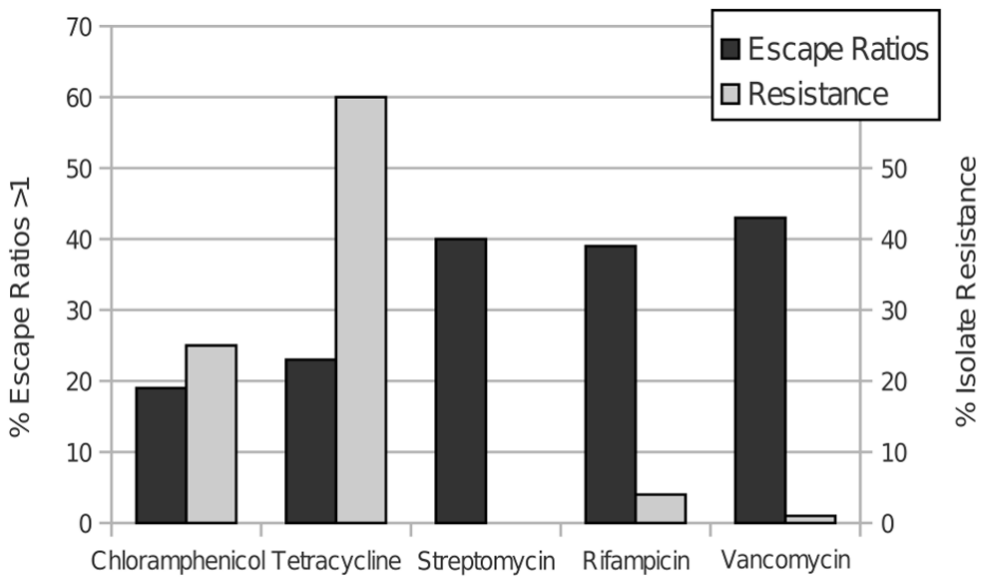
ER>1 and Resistance. Frequencies of escape ratios greater than one among all possible isolate pair-antibiotic combinations (n = 360) and frequencies of resistance to the same antibiotics among a global collection of naturally-occurring soil *Streptomyces* (21).

Among the nine isolates tested, the frequency of escape ratios >1 ranged from 5% to 68% (Figure S1). The frequency of escape ratios >1 also varied among antibiotics (Figure 3). While subinhibitory concentrations of chloramphenicol resulted in escape ratios >1 in only 19% of isolate combinations, subinhibitory concentrations of vancomycin produced escape ratios >1 in 43% of isolate combinations (Figure 3). This suggests that at subinhibitory concentrations, vancomycin often provides a potential fitness benefit to *Streptomyces* by reducing the significance of nutrient competition to growth. In contrast, chloramphenicol may reduce nutrient competition only rarely. Overall, streptomycin, rifampicin and vancomycin were significantly more likely (Chi sq, p<0.05) to enhance competition-free growth at subinhibitory concentrations than tetracycline or chloramphenicol.

The prospect that some antibiotics when present at subinhibitory concentrations can reduce the potential costs of nutrient competition to *Streptomyces* fitness has significant implications for the dynamics of antibiotic inhibitory and resistance phenotypes in soil. Specifically, if SICA can enhance fitness among coexisting competitors by reducing the costs of resource competition, there is likely to be little selection for resistance to that antibiotic. Thus, we hypothesize that there should be relatively less resistance in naturally-occurring *Streptomyces* populations to vancomycin, streptomycin and rifampicin, which reduce the significance of nutrient competition to growth in a high percentage of isolate combinations (Figure 3), than to chloramphenicol and tetracycline. Recent data [22] support this hypothesis. Within a global collection of naturally-occurring *Streptomyces* isolates, D’Costa *et al.* found relatively high frequencies of resistance to chloramphenicol and tetracycline (∼25% and 60%), while frequencies of resistance to vancomycin, rifampicin, and streptomycin, were dramatically smaller (<5%) (Figure 3). The high frequencies of resistance and correspondingly low frequencies of escape ratios >1 are consistent with the hypothesis that chloramphenicol and tetracycline act most commonly as weapons to mediate antagonistic species interactions in natural habitats. In contrast, the low frequencies of resistance in natural populations and the high frequencies of increased competition-free growth for *Streptomyces* in the presence of subinihibitory concentrations of vancomycin, streptomycin and rifampicin suggest that these antibiotics may act predominantly as signals to mediate neutral or beneficial species interactions in the environment.

Accumulating evidence suggests that antibiotics play diverse roles in natural habitats [23]–[27]. Understanding the specific roles played by different antibiotics and how they vary among ecological settings has significant implications for understanding the dynamics of selection for antibiotic inhibitory and resistance phenotypes. Our data lend support to the concept of hormesis [28], and specifically that the roles of antibiotics in mediating species interactions vary significantly depending upon whether the antibiotic is present at high vs. low concentrations in the environment. Biosynthetic pathways for antibiotic production are tightly regulated [29]–[30], often by the presence of signaling molecules–including SICA. While a simple arms race model suggests that antibiotics as weapons will impose strong selection for resistance in target populations, this work suggests that smaller concentrations of antibiotics, or antibiotics as signals, may induce a distinct coevolutionary pathway. If SICA induce shifts in nutrient use, they may enhance the likelihood of niche differentiation as a viable coevolutionary trajectory for interacting species, rather than an antibiotic arms race [12]. This may contribute to the ability of *Streptomyces* to produce a diverse array of highly-regulated antibiotics that serve distinct roles–sometimes weapons, sometimes signals– in interactions with different species, and whose production levels vary depending upon the habitat and coexisting populations. Rather than a dichotomy between weapon or signal, antibiotics appear to play a central role in mediating complex interactions within microbial communities, and the specific roles of a single antibiotic may vary across the landscape. Developing a more nuanced understanding of antibiotic-mediated species interactions in natural populations will help us effectively search for novel antibiotics, manage natural habitats to enhance inhibitory or resistant phenotypes, and develop predictive models for antibiotic-mediated coevolutionary dynamics in microbial populations.

## Supporting Information

Figure S1 **Percentage of escape ratios greater than one among** ***Streptomyces*** **isolates.** There were n = 40 isolate pair-antibiotic combinations per isolate. The frequency of escape ratios >1 varied widely among isolates (Chi Square [8,360] = 69.99 p = 0).
(TIFF)

Table S1 **Concentrations of antibiotics (µg/ml) used for each isolate-antibiotic combination.** Minimum inhibitory concentrations (MIC) were determined for each isolate-antibiotic combination on solid ISP2 medium. The concentrations defined as subinhibitory were 10% of the MIC. Growth on ISP2 containing the final subinhibitory concentration was confirmed for all isolate-antibiotic combinations.
(PDF)

Table S2 **Niche widths summarizing nutrient use of all isolates on all antibiotics.** Niche widths of each isolate in the presence and absence (CONTROL) of SICA. Treatments that differed significantly from the control are marked with an asterisk. CHLOR: chloramphenicol, STREP: streptomycin, RIF: rifampicin, VANC: vancomycin and TET: tetracycline.
(PDF)
