# Supplementary figures and images for: Subinhibitory Antibiotic Concentrations Mediate Nutrient Use and Competition among Soil Streptomyces
Source: PLoS One. 2013 Dec 5;8(12):e81064. doi: 10.1371/journal.pone.0081064 (PMC3855208; doi:10.1371/journal.pone.0081064)

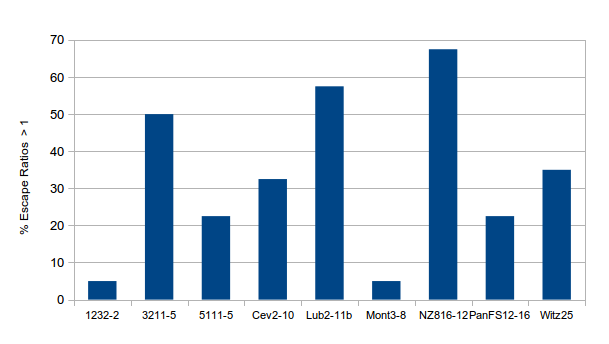

Supplement: Figure S1 — Percentage of escape ratios greater than one among Streptomyces isolates. There were n = 40 isolate pair-antibiotic combinations per isolate. The frequency of escape ratios >1 varied widely among isolates (Chi Square [8,360] = 69.99 p = 0). (TIFF) [file pone.0081064.s001.tiff]
