# Supplementary material for: Subinhibitory Antibiotic Concentrations Mediate Nutrient Use and Competition among Soil Streptomyces
Source: PLoS One. 2013 Dec 5;8(12):e81064. doi: 10.1371/journal.pone.0081064 (PMC3855208; doi:10.1371/journal.pone.0081064)
Supplement: Table S1 — Concentrations of antibiotics (µg/ml) used for each isolate-antibiotic combination. Minimum inhibitory concentrations (MIC) were determined for each isolate-antibiotic combination on solid ISP2 medium. The concentrations defined as subinhibitory were 10% of the MIC. Growth on ISP2 containing the final subinhibitory concentration was confirmed for all isolate-antibiotic combinations. (PDF) [file pone.0081064.s002.pdf]

Table S1. Concentrations of antibiotics (μg/ml) used for each isolate-antibiotic combination.

ANTIBIOTIC	1232-2	3211-5	5111-5	Cev 2-10	Lub2-11b	Mont 3-8	NZ816-12	Pan FS14	Witz 25
CHLORAMPHENICOL	2	4	4	8	1	1	0.5	8	4
TETRACYCLINE	2	2	4	1	1	1	4	2	4
STREPTOMYCIN	2	1	16	0.5	1	0.5	0.05	16	0.2
RIFAMPICIN	0.2	0.5	0.5	0.05	0.05	0.2	0.05	2	0.5
VANCOMYCIN	0.05	0.2	0.5	0.03	0.05	0.2	0.03	0.1	0.1

Minimum inhibitory concentrations (MIC) were determined for each isolate-antibiotic combination on solid ISP2 medium. The concentrations defined as subinhibitory were 10% of the MIC. Growth on ISP2 containing the final subinhibitory concentration was confirmed for all isolate-antibiotic combinations.
