# Supplementary material for: Subinhibitory Antibiotic Concentrations Mediate Nutrient Use and Competition among Soil Streptomyces
Source: PLoS One. 2013 Dec 5;8(12):e81064. doi: 10.1371/journal.pone.0081064 (PMC3855208; doi:10.1371/journal.pone.0081064)
Supplement: Table S2 — Niche widths summarizing nutrient use of all isolates on all antibiotics. Niche widths of each isolate in the presence and absence (CONTROL) of SICA. Treatments that differed significantly from the control are marked with an asterisk. CHLOR: chloramphenicol, STREP: streptomycin, RIF: rifampicin, VANC: vancomycin and TET: tetracycline. (PDF) [file pone.0081064.s003.pdf]

Table S2: Niche widths summarizing nutrient use of all isolates on all antibiotics.

	CONTROL	CHLOR	STREP	RIF	VANC	TET
1232-2	81.3	20.0 *	35.3 *	30.7 *	68.3	50.7
3211-5	58.7	49.0	70.3	63.3	80.7	66.0
5111-5	41.3	7.3 *	48.7	60.0	41.7	27.7
Cev2-10	80.0	64.0 *	69.3	75.0	60.7 *	56.3 *
Lub2-11b	69.0	57.0	51.3	58.0	85.7	69.7
Mont3-8	70.0	69.7	69.7	64.0	68.0	62.0
NZ816-12	28.3	9.7	38.0	46.3	38.0	65.0
PanFS14	48.3	41.3	25.7	47.0	50.3	19.0
Witz25	49.7	35.3	55.7	48.7	34.7	51.0

Niche widths of each isolate in the presence and absence (CONTROL) of SICA. Treatments that differed significantly from the control are marked with an asterisk. CHLOR: chloramphenicol, STREP: streptomycin, RIF: rifampicin, VANC: vancomycin and TET: tetracycline.
